# A covariate-constraint method to map brain feature space into lower dimensional manifolds

**DOI:** 10.1162/netn_a_00176

**Published:** 2021-03-01

**Authors:** Félix Renard, Christian Heinrich, Marine Bouthillon, Maleka Schenck, Francis Schneider, Stéphane Kremer, Sophie Achard

**Affiliations:** Université Grenoble Alpes, CNRS, Inria, Grenoble, France; iCube, Université de Strasbourg, CNRS, Illkirch, France; iCube, Université de Strasbourg, CNRS, Illkirch, France; Service de Médecine Intensive Réanimation, CHU de Strasbourg, France; Faculté de Médecine FMTS, Strasbourg, France; Service de Médecine Intensive Réanimation, CHU de Strasbourg, France; Faculté de Médecine FMTS, Strasbourg, France; U1121, Université de Strasbourg, France; iCube, Université de Strasbourg, CNRS, Illkirch, France; Imagerie 2, CHU de Strasbourg, Université de Strasbourg, France; Université Grenoble Alpes, CNRS, Inria, Grenoble, France

**Keywords:** Graphs, Machine learning, Connectomes, Hub disruption index

## Abstract

Human brain connectome studies aim to both explore healthy brains, and extract and analyze relevant features associated with pathologies of interest. Usually this consists of modeling the brain connectome as a graph and using graph metrics as features. A fine brain description requires graph metrics computation at the node level. Given the relatively reduced number of patients in standard cohorts, such data analysis problems fall in the high-dimension, low-sample-size framework. In this context, our goal is to provide a machine learning technique that exhibits flexibility, gives the investigator an understanding of the features and covariates, allows visualization and exploration, and yields insight into the data and the biological phenomena at stake. The retained approach is dimension reduction in a manifold learning methodology; the originality is that the investigator chooses one (or several) reduced variables. The proposed method is illustrated in two studies. The first one addresses comatose patients; the second one compares young and elderly populations. The method sheds light on the differences between brain connectivity graphs using graph metrics and potential clinical interpretations of these differences.

## INTRODUCTION

Brain modeling and understanding is a very active field of research involving different disciplines, such as neuroscience, image and signal processing, statistics, physics, and biology. These last years, neuroimaging modalities have been developed to explore the brain for both structural and functional features. It is now recognized that these images are providing very promising noninvasive observations of the brain (Bullmore & Sporns, 2009; Mwangi, Tian, & Soares, 2014; Richiardi, Achard, Bunke, & Van De Ville, 2013). One consequence of the availability of such massive datasets is the need to develop more and more sophisticated models to unravel the possible alteration of brains due to the impact of different pathologies. In this context, representing the brain as a global system is capital. This may be achieved using a *network* (Bullmore & Sporns, 2009). A brain network is a graph where nodes correspond to specific regions, and edges describe interactions and links between those regions. Different kinds of links and interactions may be of interest. Anatomical tracts are identified using diffusion imaging (Sporns, Tononi, & Kötter, 2005) and used in anatomical connectivity studies, where the whole set of links is called an *anatomical connectome*. Functional interactions are identified in functional imaging studies, whether in resting state or in task performing (Fallani, Richiardi, Chavez, & Achard, 2014; Rosazza & Minati, 2011), and used in functional connectivity studies. The whole set of functional links is called a *functional connectome*. In the functional case, brain networks are particularly adequate in encapsulating both spatial and temporal information in a single model. Indeed, brain networks are constructed using brain parcellation, namely spatial features, and time series interactions, namely temporal features. This model has attracted lots of attention these last 20 years by providing both very intuitive and spatial maps of brain networks.

Brain networks can be quantified using graph metrics such as minimum path length, clustering (Watts & Strogatz, 1998), global and local efficiency (Latora & Marchiori, 2001), modularity (Newman, 2006), and assortativity (Newman, 2002), among others. As these metrics are associated with specific network features, it is often possible to find the appropriate metrics to use given specific neuroscience hypotheses of the study. For the study of brain disorders, these metrics have been used in order to extract biomarkers for pathologies such as for example Alzheimer’s disease (Supekar, Menon, Rubin, Musen, & Greicius, 2008), schizophrenia (Lynall et al., 2010), and multiple sclerosis (Filippi et al., 2014). Extracting quantitative parameters of brain networks is compulsory to conduct any statistical analysis. In this framework, statistical and machine learning approaches on graph metrics on all nodes allow the quantification of differences between groups (Richiardi et al., 2013).

For any dataset, any graph metric can be computed either at the global level with one value for an entire network or at the nodal level with one value for each node and a vector of values for the entire network. It has already been shown that global values may not discriminate two groups of subjects (Achard et al., 2012), which shows their limits as biomarkers. Few attempts have been made to directly use distances between networks such as the edit distance (Mokhtari & Hossein-Zadeh,^2013^), or network similarities (Mheich et al., 2017). However, nodal-level approaches are challenging since hundreds of brain areas can be extracted whereas the number of subjects is generally small. This corresponds to the high-dimension, low-sample-size (HDLSS) configuration and falls under the curse of dimensionality (Bellman, 1961). In particular, standard classification and regression algorithms are not robust anymore in such a context (Hastie, Tibshirani, & Friedman, 2001, Chapter 2, Section 5, and Chapter 18).

Dimension-reduction techniques tackle curse of dimensionality issues (Hastie et al., 2001). In this framework, feature selection, where a subset of the original variables is considered, and feature extraction, where the original variables are transformed to a smaller set, may be envisaged (Webb, 2002). We resort here to the ISOMAP methodology, which is a well-known nonlinear feature extraction algorithm generalizing principal component analysis (PCA) dimension reduction (Huo, Ni, & Smith, 2007; Tenenbaum, de Silva, & Langford, 2000). ISOMAP may be seen as a manifold learning approach, where the degrees of freedom of the data are captured by the latent variables, and where the structure of points in the latent space (the reduced space) mimics the structure of data in the original space. Nevertheless, ISOMAP raises two issues: interpreting the latent variables and determining the effect a change in the latent variables incurs in the data space; that is, the corresponding changes in brain networks and the underlying neuroscience hypotheses at stake in the case of the present study.

Dimension reduction is not new in the field of brain connectivity studies. Several methods have been proposed to extract nodal features at the level of brain regions. Using the hub disruption index (the *κ* index) to analyze a set of brain networks may be considered a feature extraction approach: This is a user-defined transformation of the original space to a 1D latent space (Achard et al., 2012). Principal component analysis was previously applied on graph metrics in Robinson, Hammers, Ericsson, Edwards, and Rueckert (2010) with vectors representing brains at the nodal level. We proposed in Renard, Heinrich, Achard, Hirsch, and Kremer (2012) to use kernel PCA, a nonlinear version of PCA. Moreover, interpreting latent variables may be addressed by correlating the reduced space with clinical data (Gerber, Tasdizen, Thomas Fletcher, Joshi, & Whitaker, 2010). Covariates may also be mapped or regressed on the reduced space as proposed in Aljabar, Wolz, and Rueckert (2012), thus shedding light on latent variables. Dimension-reduction methods have also been applied to connectivity matrices (Ktena et al., 2018; Kumar, Toews, Chauvin, Colliot, & Desrosiers, 2018; Yamin et al., 2019) or to the voxel time series (Saggar et al., 2018), mainly for classification purposes. It is indeed difficult using the whole connectivity matrices or voxel time series to give an interpretation at the nodal or voxel level (Gallos & Siettos, 2017; Haak, Marquand, & Beckmann, 2018; Laurienti et al., 2019). Network embedding framework can be viewed as a dimension-reduction method and was also applied to brain connectivity graphs (Rosenthal et al., 2018).

The objective of this article is to integrate all features cited above in one method: working at the nodal level, applying dimension-reduction techniques, and mapping covariates to ease interpretation. In addition, a new methodology is proposed to incorporate interesting network features already identified in specific datasets directly in the manifold learning approach. Contrary to statistical tests at nodal levels where each feature is treated independently of others, our approach based on machine learning is able to analyze joint variations between local descriptors.

This paper is focusing on two already published datasets. The first one consists of fMRI datasets on 20 healthy controls and 17 coma patients from Achard et al. (2012). The second one is based on Achard and Bullmore (2007), where 15 young healthy subjects and 11 elderly healthy subjects were scanned using resting-state fMRI. Our first experiment compares data-driven approaches such as linear discriminant analysis (LDA) and random forests (RF) to an ad hoc description such as the hub disruption index *κ*. This allows us to compare classical machine learning approaches, in which the interpretability of the results is often difficult, with approaches resorting to descriptors constructed using neuroscientific hypotheses. This first experiment can be seen as preliminaries of the sequel of the paper, where a feature is extracted for each individual in order to optimize classification of the two groups using either classical machine learning approaches or ad hoc descriptors. The second experiment consists of constructing a data-driven manifold, ISOMAP, using the graph metrics as features. ISOMAP is providing a compact representation of brain connectomes in a reduced space, where it is straightforward to map the available covariates. In addition, we may interpret changes in connectomes by regressing covariables like *κ* on the reduced space using latent variables.

This representation allows a visualization of each subject relative to the whole population, which is crucial in clinical studies, for example in order to better understand brain changes for each specific subject. In addition, *κ* has been shown to be both a meaningful descriptor and a good classifying feature for brain connectomes of coma patients. Therefore, we propose a new method based on a covariate-constrained manifold learning (CCML) using *κ* as an input of ISOMAP. This allows us to propose a new generative model based on our new data representation, to better predict the variation in each patient given the changes of covariables. Based on the results of the first experiment, the choice of the covariate, *κ* in this work, can be adjusted to the studied datasets.

## MATERIALS AND METHODS

### Resting-State fMRI Data

#### Comatose study.

The data were acquired in a previous study aimed at characterizing resting-state connectivity brain networks for patients with consciousness disorders. The description of the data and results is reported in Achard et al. (2012). The patients were scanned a few days after major acute brain injury, when sedative drug withdrawal allowed for spontaneous ventilation. Therefore, all patients were spontaneously ventilating and could be safely scanned at the time of fMRI. The causes of coma are patient-dependent: 12 had cardiac and respiratory arrest due to various causes; 2 had a gaseous cerebrovascular embolism; 2 had hypoglycemia; and 1 had extracranial artery dissection. A total of 25 patients were scanned (age range, 21–82 years; 9 men). Data on eight patients were subsequently excluded because of unacceptable degrees of head movement. The coma severity for each patient was clinically assessed using the 62 items of the WHIM scale: scores range from 0, meaning deep coma, to 62, meaning full recovery. Six months after the onset of coma, 3 patients had totally recovered, 9 patients had died, and 5 patients remained in a persistent vegetative state. The normal control group is composed of 20 healthy volunteers matched to the group of patients for sex (11 men) and approximately for age (range, 25–51 years). This study was approved by the local Research Ethics Committee of the Faculty of Health Sciences of Strasbourg on October 24, 2008, (CPP 08/53) and by the relevant healthcare authorities. Written informed consent was obtained directly from the healthy volunteers and from the next of kin for each of the patients. Resting-state data were acquired for each subject using gradient echo planar imaging technique with a 1.5-T MR scanner (Avanto; Siemens, Erlangen, Germany) with the following parameters: relaxation time = 3 s, echo time = 50 ms, isotropic voxel size = 4 × 4 × 4 mm^3^, 405 images, and 32 axial slices covering the entire cortex. The preprocessing of the data is detailed in our previous study (Achard et al., 2012).

#### Young and elderly study.

The data used in this study have already been analyzed in two papers (Achard & Bullmore, 2007, and Meunier, Achard, Morcom, & Bullmore, 2009). The goal of these papers was to identify the changes in brain connectomes for elderly subjects in terms of topological organization of brain graphs. The data consist of 15 young subjects aged 18–33 years, mean age = 24 and 11 elderly subjects aged 62–76 years. Each subject was scanned using resting-state fMRI as described in Achard and Bullmore (2007, Wolfson Brain Imaging Centre, Cambridge, UK). For each dataset, a total of 512 volumes were avalaible with number of slices, 21 (interleaved); slice thickness, 4 mm; interslice gap, 1 mm; matrix size, 64 × 64; flip angle, 90°; repetition time (TR), 1,100 ms; echo time, 27.5 ms; in-plane resolution, 3.125 mm.

### Preprocessing and Wavelet Graph Estimation

Brain network graphs were determined following Achard et al. (2012) for the comatose study and Achard and Bullmore (2007) for the young and elderly study. For each subject, data were corrected for head motion and then coregistered with each subject’s T1-weighted structural MRI. Each subject’s structural MRI was nonlinearly registered with the Colin27 template image. The obtained deformation field image was used to map the fMRI datasets to the automated anatomical labeling (AAL) or to a customized parcellation image with 417 anatomically homogeneous size regions based on the AAL template image (Tzourio-Mazoyer et al., 2002). Regional mean time series were estimated by averaging the fMRI time series over all voxels in each parcel, weighted by the proportion of gray matter in each voxel of the segmented structural MRIs. We estimated the correlations between wavelet coefficients of all possible pairs of the *N* = 90 or 417 cortical and subcortical fMRI time series extracted from each individual dataset. For the coma, only scale 3, 0.02–0.04 Hz, wavelet correlation matrices were considered. For the young and elderly, the wavelet scale considered corresponds to 0.06–0.11 Hz. The choice of these wavelet scales or frequency bands is explained precisely in the corresponding papers (Achard & Bullmore, 2007; Achard et al., 2012). To generate binary undirected graphs, a minimum spanning tree algorithm was applied to connect all parcels. The absolute wavelet correlation matrices were thresholded to retain 2.5% of all possible connections. Each subject was then represented by a graph with nodes corresponding to the same brain regions, and with the same number of edges.

### Graph Metrics

The objective is to extract differences between the two groups with respect to the topological organization of the graphs. Each graph is summarized by graph metrics computed at the nodal level. Three metrics are considered here: degree, global efficiency, and clustering (Bullmore & Sporns, 2009).

The degree is quantifying the number of edges belonging to one node. Let *G* denote a graph with *G*_*ij*_ = 0 when there is no edge between nodes *i* and *j*, and *G*_*ij*_ = 1 when there is an edge between nodes *i* and *j*. The degree *D*_*i*_ of node *i* is computed as

$$D_{i}=\underset{j\in G,j\neq i}{\sum }G_{ij}.$$ (1)

The global efficiency measures how the information is propagating in the whole network. A random graph will have a global efficiency close to 1 for each node, and a regular graph will have a global efficiency close to 0 for each node. The global efficiency *Eglob* is defined as the inverse of the harmonic mean of the set of the minimum path lengths *L*_*ij*_ between node *i* and all other nodes *j* in the graph:

$$Eglob_{i}=\frac{1}{N-1}\underset{j\in G}{\sum }\frac{1}{L_{ij}}.$$ (2)

Clustering is a local efficiency measure corresponding to information transfer in the immediate neighborhood of each node, defined as

$$Clust_{i}=\frac{1}{N_{G_{i}}(N_{G_{i}}-1)}\underset{j,k\in G_{i},j\neq k}{\sum }\;\frac{1}{L_{jk}},$$ (3)

where *G*_*i*_ is the subgraph of *G* defined by the set of nodes that are the nearest neighbors of node *i*. A high value of clustering corresponds to highly connected neighbors of each node, whereas a low value means that the neighbors of each node are rather disconnected.

**Figure 1.**
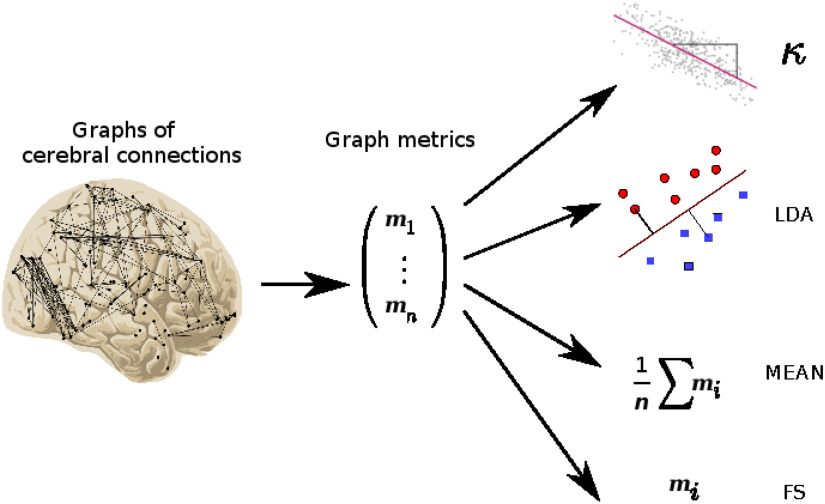
General framework from graphs of cerebral connectomes to the different scalar features. Brain connectivity graphs are extracted from fMRI data. Graph metrics are computed at the nodal level for each subject. The matrices of graph metrics can then be analyzed using different methods: the hub disruption index based on regression analyses (*κ*); linear discriminant analysis (LDA); average of metrics (MEAN); and feature selection (FS). Each of these methods allows us to summarize the graph metric in one scalar for each subject in order to better differentiate the studied populations.

Each graph metric emphasizes a specific property at the nodal level. With a view to statistical comparison, several methods have already been developed, representing data in specific spaces. Each method aims to separate classes. Usually these methods are very general and can be applied without careful inspection of the data. We used here four different methods (Richiardi, Achard, Bullmore, & Van De Ville, 2011): the *κ* index resulting from a careful inspection of the data, mean over graph metrics (denoted here MEAN), LDA, and feature selection (FS) by selecting the best feature based on a univariate statistical student *t* test. Like the *κ* index, each of these methods provides, for each patient, a scalar feature corresponding to a particular property of the data. Figure 1 gives an illustration of the different methods.

### *κ* Index Definition

**Figure 2.**
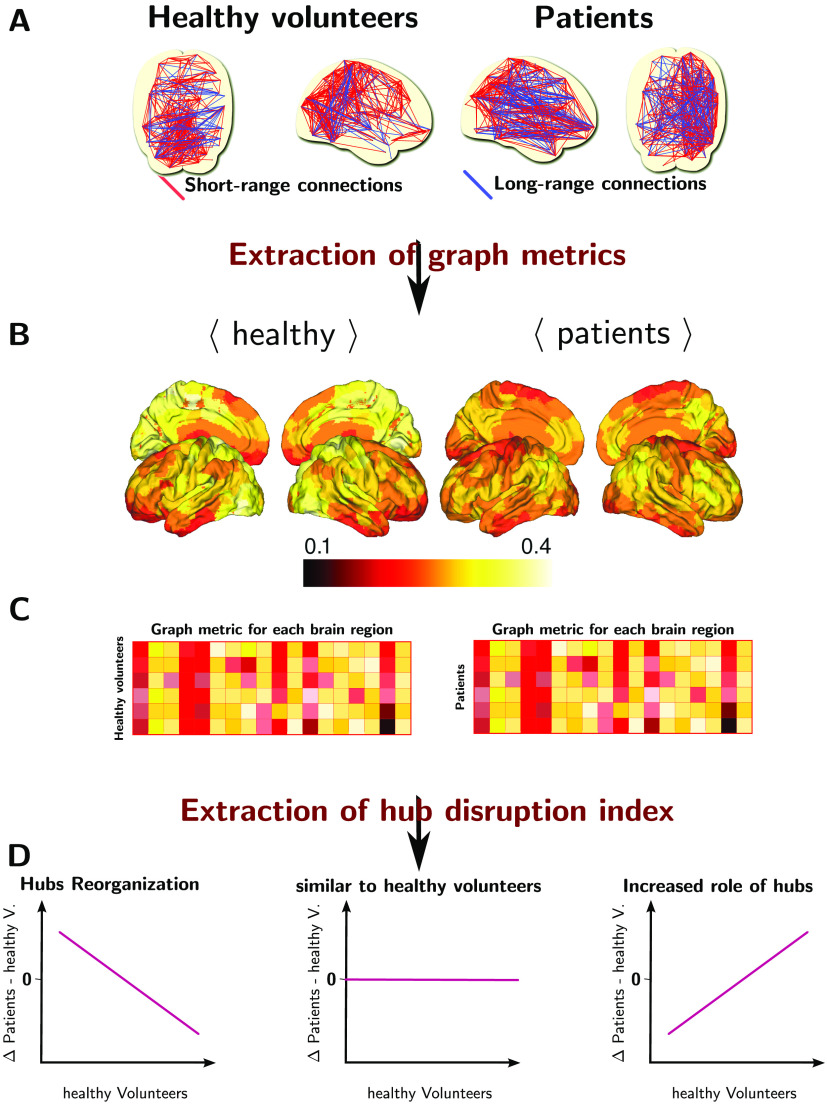
Extraction of hub disruption index *κ*. (A) Brain connectomes inferred for each subjects. (B) For each brain connectome, extraction of graph metrics for each region of the brain. (C) Matrix representation of the graph metrics, where a row corresponds to a subject and a column corresponds to a brain region. (D) Computation of the hub disruption index by regressing the average of brain metrics of the difference of patients and average of healthy volunteers against the average of healthy volunteers. The hub disruption index corresponds to the slope coefficient. We give several illustrations following the sign of this coefficient.

In our previous study (Achard et al., 2012), *κ* was devised to compare graph metrics obtained on each node of a subject or of a group with a reference group (see Figure 2). In classical comparisons between a group of patients and a group of healthy volunteers, the reference is the group of healthy volunteers. In the present study, for a given graph metric and two groups, we first compute the average of this metric for each node over the group of healthy volunteers, denoted as the reference. Each subject is then summarized as a vector of values of dimension of the number of nodes. Then, for each patient, *κ* corresponds to the slope of the regression of a nodal graph metric between the given patient minus the reference and the reference. Let *N* denote the number of nodes in the graph, *n*_*p*_ the number of patients, and *n*_*c*_ the number of controls. Let (*m*_1_, …, *m*_*n*_*p*__) ∈ ℝ^*N*×*n*_*p*_^ denote a matrix of graph metric extracted given the graphs of patients, for *j*, 1 ≤ *i* ≤ *n*_*p*_, *m*_*j*_ ∈ ℝ^*N*^. For each *j*, *m*_*j*_ is equal to one graph metric such as *D*, *Eglob*, or *Clust*. Let us also define a similar matrix for the controls, (*h*_1_, …, *h*_*n*_*c*__) ∈ ℝ^*N*×*n*_*c*_^. Let us define the average metric for controls, for each *i*, 1 ≤ *i* ≤ *N*,

$$\overset{-}{h_{i}}=\frac{1}{n_{c}}\overset{n_{c}}{\underset{j=1}{\sum }}h_{ij}.$$ (4)

*κ* is defined by the following regression:

$$m_{i}-h_{i}=\kappa {\overset{-}{h}}_{i}+\epsilon_{i},$$ (5)

where *ϵ*_*i*_ is the classical error term in linear regression. In order to give a simple interpretation of *κ*, we assume that the global graph metric computed as an average over the nodes is the same in both groups. A value of 0 for *κ* is showing that the graph metric obtained at the node level is the same for the patient and the reference. A positive value of *κ* is indicating that the hubs and non-hubs of the patient in comparison to the reference are located on the same nodes. However, the values of the graph metrics are increased for the hubs and decreased for the non-hubs. Finally, when the value of *κ* is negative, the hubs of the reference are no longer hubs of the patient, and the non-hubs of the reference are hubs for the patient. In Achard et al. (2012), we showed that the *κ* index is able to discriminate both groups (coma patients and healthy volunteers) while the global metric is unable to identify any significant difference. Instead of averaging the graph metrics, the *κ* index is capturing a joint variation of the metrics computed for each node.

### Mean Over the Nodes (MEAN)

For each graph metric, the mean over the nodes of the graph captures a global property of the network. These global metrics have been previously used to discriminate two populations of networks, for example for Alzheimer’s disease (Supekar et al., 2008) and for schizophrenia (Lynall et al., 2010). Such a coefficient can discriminate two networks well when their topologies are really different. However, such metrics do not take into account the specificity of the nodes. Indeed, when permuting the nodes of the graph, the global metric is not changed, but the hubs of the graph are not associated with the same nodes anymore. Therefore, a graph reorganization cannot be detected using such global metrics.

### Linear Discriminant Analysis (LDA)

LDA (Fisher, 1936) is a classification method that aims to aiming at identify the linear projection optimally separating two groups. It can be considered a gold standard for linear group discrimination. It is not specific to the analysis of networks.

LDA has been previously used for network discrimination in Robinson et al. (2010). This algorithm amounts to computing a scalar for each graph. However, there is no simple clinical interpretation of the discriminating parameter.

### Feature Selection (FS)

As for LDA, FS determines the features yielding the best separation of the two groups. Several features may be used simultaneously. In order to establish a fair comparison with the other methods, we choose to extract the single feature yielding the best separation. Several methods exist for FS. We choose univariate FS implemented in Pedregosa et al. (2011). An advantage of FS is that it is capturing discriminative features at the node level. As the selected features are extracted directly from the data, it is usally possible to derive a clinical interpretation. However, joint variations are not modeled, and on the comatose study FS is not able to yield results of the same quality as those obtained using *κ*.

### Modeling Populations of Networks with Manifold Learning

ISOMAP (Tenenbaum et al., 2000) is used as a manifold learning approach to describe population networks. We propose an original approach based on ISOMAP, where we constrain one variable of the reduced space (the latent space) to correspond to a covariate.

#### Manifold learning using ISOMAP.

ISOMAP devises a reduced-dimension version of the original set of points. Interpoint distances in the reduced space reproduce as much as possible interpoint distances in the original space. Euclidean and geodesic distances are respectively used. Principal component analysis may be seen as a particular case of ISOMAP, where Euclidean distances are used in the original space, instead of geodesic distances. The reader is referred to Tenenbaum et al. (2000) for details about the algorithm.

In our case, the original data correspond to a vector of graph metrics for each subject, the dimension of the vector being the number of nodes times the number of metrics. For each analysis, only one metric is considered here. However, this method could be applied by jointly using several metrics. Covariates may be regressed on the reduced space. In the present work, this was achieved using a classic radial basis function interpolation.

The choice of the ISOMAP is twofold: First, the estimated reduced space is a smooth manifold, and preserves the global structure of the dataset. Notably, the reduced space exhibits a continuum function of subjects. Second, the cost function of the ISOMAP allows the integration of additional constrained scores. ISOMAP was performed by computing a nearest neighbor graph connecting the four nearest neighbors according to the Euclidean distance. This distance correctly reflects the local topology of the graph metrics space. The choice of four neighbors is driven by the relatively small number of subjects in the study.

The classification score of the ISOMAP was computed using a nonlinear support vector machine (SVM) approach with radial basis function kernel in the reduced space (Hearst, Dumais, Osuna, Platt, & Scholkopf, 1998).

#### Covariate constrained manifold learning.

One drawback of manifold learning algorithms is the difficulty of interpreting the reduced coordinates because they are usually meaningless. The original method proposed in this work consists of constraining one coordinate of the reduced space to correspond to a specific covariate. The other coordinates are left unconstrained, as in classical ISOMAP. Such a procedure requires special care regarding the optimization aspect. We apply a strategy proposed in Brucher, Heinrich, Heitz, and Armspach (2008), where points are introduced one by one.

Moreover, a scale factor *α* is considered for the axis corresponding to the covariate. This parameter, obtained by optimization, balances the scales of the different axes.

The reduced point ${\tilde{x}}_{i}$ is defined by ${\tilde{x}}_{i}$ = [*αc*_*i*_; **x**_*i*_]^*T*^, where *c*_*i*_ is the chosen covariate and **x**_*i*_ are the other coordinates. The cost function *E* is defined as

$$E=\underset{i,i<j}{\sum }{\left(||{\tilde{x}}_{i}-{\tilde{x}}_{j}|{|}^{2}-||y_{i}-y_{j}|{|}^{2}\right)}^{2},$$ (6)

where {**y**_*i*_}_*i* =1,…,*N*_ is the graph metric vectors over *N* graph nodes. For an incoming data point *i*, the cost function *E* is optimized three times with regard to (a) **x**_*i*_ as $\underset{x_{i}}{\min}$ *E*, (b) *α* as $\underset{\alpha }{\min}$ *E*, and (c) **x**_*j*_ for each point that has already been included as $\underset{{\left\{x_{j}\right\}}_{j=1,\ldots ,i-1}}{\min}$ *E*. We consider *i* < *j* in the sum of the cost function to avoid counting the errors between two samples twice.

The distance in the cost function is the Euclidean one. Since the samples are added sample by sample, this distance reflects only the local neighborhood of the new added one.

To facilitate optimization and to avoid possible local minima, instead of inserting the samples at random, we choose the sample to be incorporated next as the one with the largest geodesic distance to the samples already incorporated. Indeed, interpolation problems are always easier than extrapolation problems where greater uncertainty may occur. We initialize the procedure by taking the two samples with the largest geodesic distance. The first two samples are used as landmarks of the border of the reduced space, and the insertion of new samples will generate only small displacements of the already inserted samples.

The algorithm is described in Algorithm 1 and available at https://github.com/renardfe/CCML (Renard, 2020).


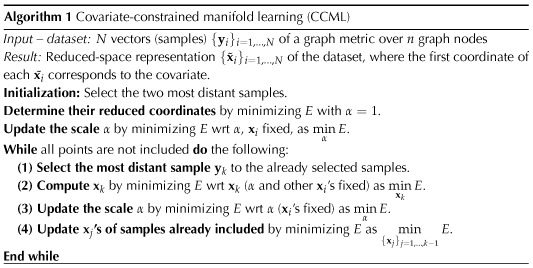


### Application: A Generative Model for the Prediction of the Variation in a Subject with Regard to the Changes of a Covariate

From the obtained embedding, a generative model

$$\hat{y}=f(\tilde{x})$$ (7)

can be devised, where $\hat{y}$ is a vector in the original space (the connectome space), $\tilde{x}$ is a vector from the manifold embedding, and *f* is a regression function. Multivariate adaptive regression splines (MARS) (Friedman, 1991) is chosen for the regression function *f* for its nice properties (one regression for each coordinate of *f*, i.e., *n* regressions): locally linear and globally nonlinear. The parameters of *f* can be determined using the dataset {**y**_*i*_}_*i* =1,…,*N*_ and the corresponding reduced vectors {${\tilde{x}}_{i}$}_*i*=1,…,*N*_ using the following equation:

$$y_{i}=f({\tilde{x}}_{i})+\epsilon_{i}={\hat{y}}_{i}+\epsilon_{i},$$ (8)

where ***ϵ***_*i*_ is the residual between a sample and its prediction ${\hat{y}}_{i}$. The residuals allow us to evaluate the accuracy of the regression function.

This kind of model is not original, PCA being the most well-known case where the model is defined as **y** = **A** $\tilde{x}$ + ***ϵ*** (see, e.g., Lawrence, 2004; Sfikas & Nikou, 2016, for references). Such a generative model used in the CCML framework allows us to determine changes in the original space (the connectome space) generated by a displacement in the reduced space, for example along the covariate axis.

## RESULTS

The different algorithms have been implemented in the Python language using the scikit-learn toolbox (Pedregosa et al., 2011). When left unspecified, coma data are used. The use of the young and elderly data is explicitely stated.

### Local Analysis Using Dimension Reduction

Permutation tests are performed on the *κ* index and on the three other measures (LDA, FS, MEAN) to assess the ability of those four metrics to discriminate two populations. More precisely, for each coefficient separately, the difference of the means of the two populations is determined for the observed populations. The labels of the samples are then shuffled and the difference of the means of the shuffled two populations is determined. This latter step is performed 10^4^ times. This method allows us to avoid making any assumptions on the distribution of the statistic. Simultaneously, the correlations between the observed *κ* index and the other coefficients are estimated.

The results corresponding to the different methods that aim to discriminate the two groups (control and coma) are given in Table 1. As expected, the machine learning algorithms (i.e., *LDA* and *FS*) show good performances in separating the two groups for different graph metrics. This is consistent with the fact that these methods have been tailored to classify the two groups. The results with the *κ* index show similar performances in separating the two groups. The large correlations between machine learning algorithms on the one hand and the graph metric *κ* on the other hand show retrospectively that similar performances were to be expected.

**Table 1:** *P* value of permutation tests comparing the mean of the two groups (10^4^ permutations, which bounds the *p* values). The correlation scores are estimated between the *κ* index and the three other measures (*LDA*, *FS*, and *MEAN*).

Mean diff. or correlation	*Eglob* (*p* value)	corr. (*p* value)	*Clust* (*p* value)	corr. (*p* value)	*D* (*p* value)	corr. (*p* value)
*κ*	0.79 (< 10^−4^)		0.75 (< 10^−4^)		0.81 (< 10^−4^)	
*LDA*	−2.89 (< 10^−4^)	0.88 (< 10^−4^)	−1.78 (< 10^−4^)	0.87 (< 10^−4^)	−2.39 (< 10^−4^)	0.88 (< 10^−4^)
*FS*	0.12 (< 10^−4^)	0.60 (10^−4^)	−0.50 (< 10^−4^)	−0.66 (10^−4^)	21.93 (< 10^−4^)	0.60 (10^−4^)
*MEAN*	0.14 (0.58)	0.25 (0.78)	−0.01 (0.43)	−0.19 (0.85)		

Moreover, a strong relationship can be observed between *κ* and LDA (correlation scores greater than 0.87 for each metric). The FS correlation scores are lower than the LDA correlation ones. The difference between the two methods is that LDA considers a linear combination of features, with a global perspective, whereas FS selects one feature and acts locally. Since *κ* reflects a global reorganization of the brain, it is expected that the correlation score of LDA will be greater than the FS one. Finally, MEAN scores reveal that this measure is not appropriate in this study.

### Standard ISOMAP Manifold Learning and the *κ* Index

In this section, the goal is to link the reduced space obtained by manifold learning to different covariates such as the *κ* index. We want to assess whether a given covariate varies smoothly across the reduced space, and is therefore predictible using this space.

**Figure 3.**
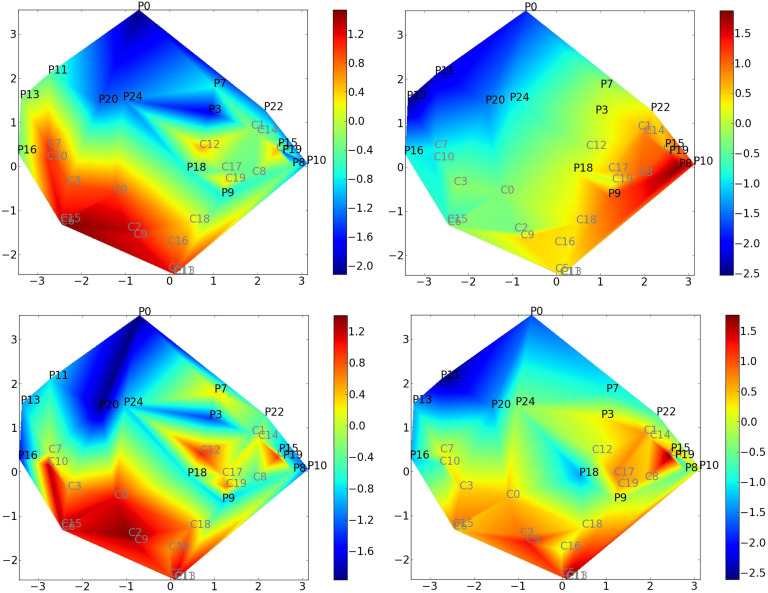
Standard ISOMAP reduced-space representation of the original dataset. *Pi*: (comatose) patient *#i*; *Cj*: control *#j*. Covariates are mapped onto the reduced space (covariate value is color coded). Top left: *κ* index mapping; top right: MEAN mapping; bottom left: LDA mapping; bottom right: FS mapping.

Figure 3 represents the reduced space obtained using standard ISOMAP, as opposed to using CCML. The values of the different covariates are color coded. The reduced-space representation allows us to separate both populations. By visual inspection of the color-coded maps, it appears that those regression maps are capturing features corresponding to *κ* and MEAN.

In order to quantify these visual observations, covariates are regressed on the reduced space. The root mean square error (RMSE) and the maximum error *M* are determined in a leave-one-out procedure. The results are given in Table 2. It can be noted that the MEAN strategy gives the same values for the graph metric degree D for all graphs since the number of edges is set to be the same for all graphs.

**Table 2.** Assessment of the regression of covariates on the reduced space. Three different reduced spaces are at stake, one for each graph metric. Root mean square error (RMSE) and maximal error *M* are displayed. The MEAN strategy is not relevant for the degree *D* since the degrees *D* for all graphs are equal (the number of edges is set to be the same for all graphs).

Covariate	*Eglob* RMSE	*Eglob* M	*Clust* RMSE	*Clust* M	*D* RMSE	*D* M
*κ*	0.51	2.44	0.68	2.59	0.26	2.14
*LDA*	0.97	7.97	0.90	5.44	0.66	4.13
*FS*	0.64	2.20	1.10	9.12	0.83	7.49
*MEAN*	0.15	0.73	1.02	5.44		

In this table, the lower the values, the better the adequacy with the reduced space. It appears that *κ* is the best choice across all metrics, except for *Eglob* where it is outperformed by MEAN. In the case of *Eglob*, this suggests that both *κ* and MEAN scores correspond to degrees of freedom of the intrinsic manifold of the functional connectivity graphs.

**Figure 4.**
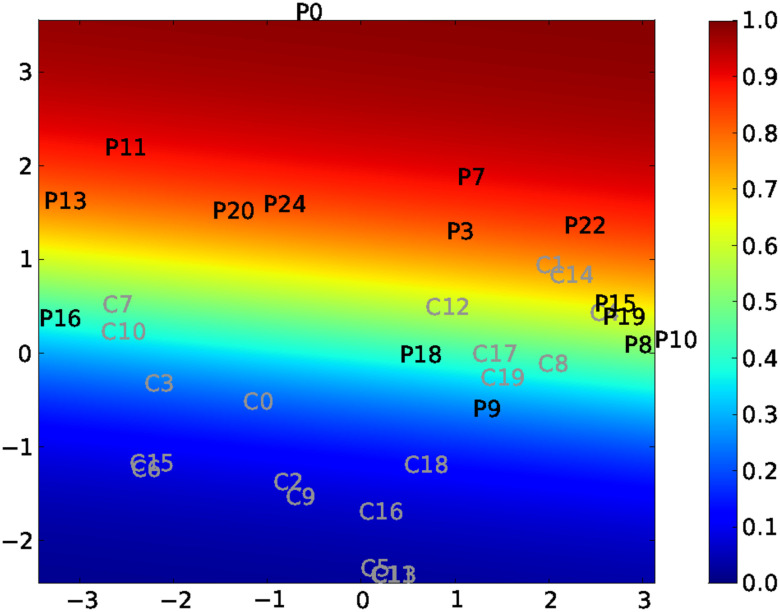
Logistic regression using reduced space stemming from standard ISOMAP. The color codes the probability of belonging to the comatose class.

Figure 4 displays the probability of belonging to a specific class computed using logistic regression on the reduced space stemming from standard ISOMAP. The probability of belonging to the comatose class is color coded in Figure 4.

This probability estimation using logistic regression is compared with covariates such as *κ* or MEAN, in Table 3. A high correlation score is observed between *κ* and logistic regression probablity. The correlation score between the MEAN coefficient and the probabilistic mapping is lower than the one with *κ*, as expected.

**Table 3.** Correlation scores between the probabilistic mapping and the different coefficients (*κ* index and MEAN measure). A high correlation score of the *κ* index indicates a good fitting between the reduced-space representation and the classification of the two groups. The *p* values are all smaller than 10^−12^.

Coefficients	*Eglob*	*Clus*	*D*
*κ*	0.87	0.87	0.81
*MEAN*	0.55	0.42	

Taken together, these observations demonstrate the importance of *κ* in the classification of these populations. Obviously, for the special case of the global efficiency metric, the MEAN score correctly describes the reduced space, but it does not correspond to the classification pattern.

### Covariate-Constrained Manifold Learning

#### Comatose population.

First we evaluate the convergence of the optimization problem (Algorithm 1). To assess the difficulty of the optimization problem, we ran it with 500 random initializations. Only 63% of the runs converged to the same solution, whereas 37% of the runs converged to a local (worse) optimum. It thus appears that our initialization procedure addresses the local optimum issue. Nevertheless, this optimization problem would probably deserve further investigation, which is outside the scope of this paper.

**Figure 5.**
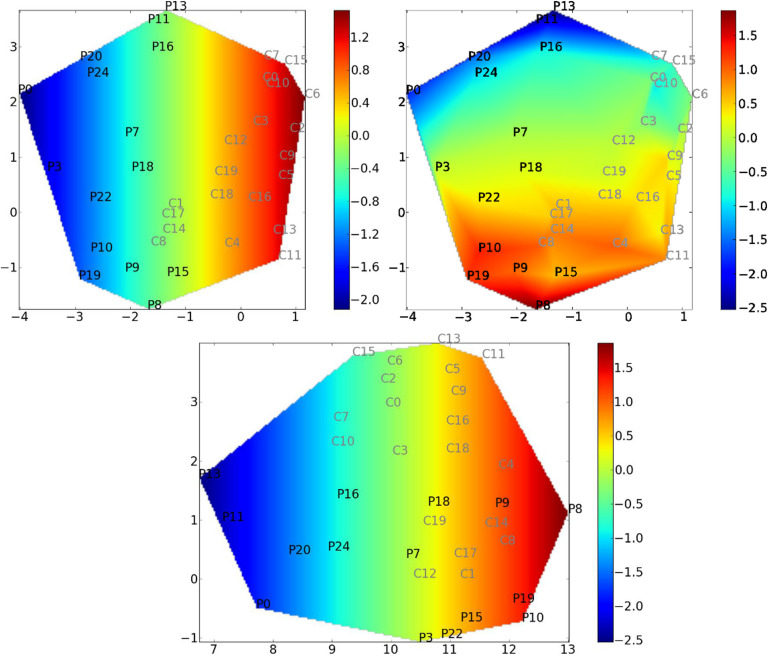
Covariate mapping onto the reduced space given by our method, CCML, using global efficiency as a graph metric. The reduced space is computed using *E*_*glob*_ as a graph metric (the *y*_*i*_’s). Covariate value is color coded. For each subfigure, the coordinates correspond to [*αc*_*i*_; *x*_*i*_]^*T*^, where *c*_*i*_ is the constrained variable and *x*_*i*_ is the free parameter. Top left: *κ* mapping with a *κ*-constrained reduced space. Top right: MEAN mapping with a *κ*-constrained reduced space. Bottom: MEAN mapping with a MEAN-constrained reduced space. As expected, we can observe that the mappings correlate well with the first coordinate by construction (top left and bottom). It is also clear that using a *κ*-constrained reduced space is facilitating the discrimination between the two populations. Indeed, the controls and patients are not covering the same part of the reduced space. On the contrary, as expected using the MEAN-constrained reduced space, the method is not providing a very clear discrimination between patients and controls. Especially, patients 9 and 18 are very close to controls. Finally, the top right figure is showing a correlation between the second coordinate of CCML and the MEAN.

In Figure 5, we display the reduced space corresponding to our new manifold learning algorithm. We can observe that the two populations are well discriminated in the case of *κ*, but not for the MEAN coefficient. This is quantified by applying a classical SVM procedure in the reduced space. We obtained the following results: for CCML, 1; for ISOMAP, 0.86; for LDA, 0.91; for *κ*, 0.89; and for MEAN and FS, 0.57.

We observe strong interaction between *κ* and the MEAN in the top right of Figure 5, where the reduced space on one axis is *κ* and the mapping corresponds to the MEAN. To quantify this, in the case of *κ*, we estimate the correlation between the second coordinate and the MEAN score. The obtained score equals 0.92, which confirms the intrinsic relationship between *κ* and the MEAN coefficient.

#### Elderly and young population.

The elderly and young groups are investigated in this section.

**Figure 6.**
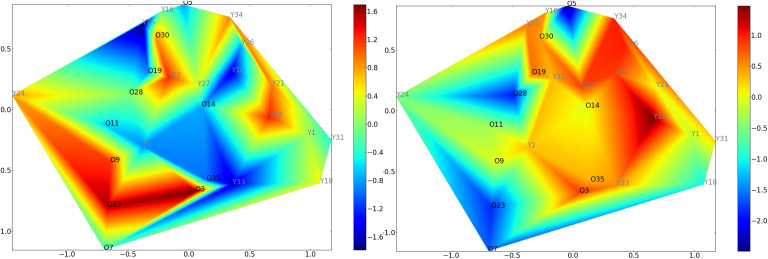
Covariate mapping onto the standard ISOMAP reduced space using global efficiency as a graph metric. Left: *κ* mapping with the standard ISOMAP reduced space. Right: MEAN mapping with the same reduced space using global efficiency as a graph metric. The old controls (resp. young controls) are labeled O (resp. Y). For these groups, the *κ* index cannot discriminate the two groups. However, the MEAN index behaves better for the discrimination between the two groups. In each case, the interpretation is complex since the mapping of the covariate is not linear.

Figure 6 displays the manifold obtained by standard ISOMAP. It is interesting to highlight that the *κ* index is not a pertinent feature to discriminate the old from the young, whereas the MEAN is a better discriminating feature. In both cases, the interpretation of the mapping is complex since it is not smooth.

**Figure 7.**
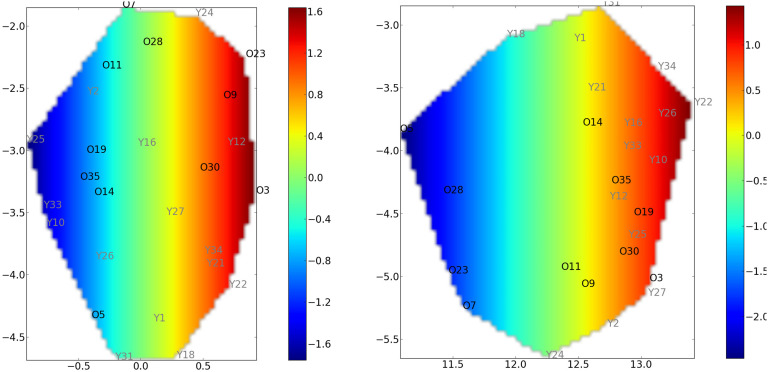
CCML approach using global efficiency as graph metric. Left: *κ* mapping with the *κ*-constrained reduced space. Right: MEAN mapping with the MEAN-constrained reduced space. The old controls (resp. young controls) are labeled O (resp. Y). Two manifolds (one for each constraint) have been determined. However, only the MEAN-constrained manifold discriminates both groups.

Figure 7 displays results from CCML for the MEAN coefficient. We can observe that the MEAN mapping discriminates the two groups.

### Application: A Generative Model for the Prediction of the Variation in a Subject with Regard to the Changes of a Covariate

**Figure 8.**
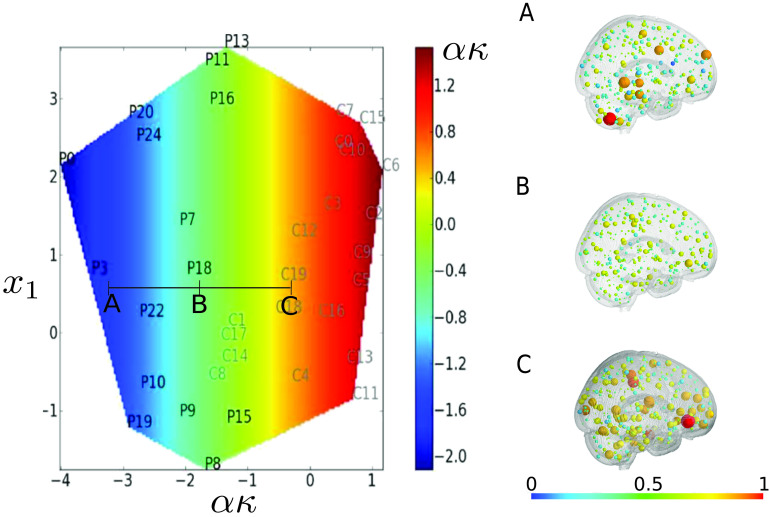
Variation in one patient along the covariate axis using global efficiency as a graph metric. The size and the color of the nodes on the right part of the figure are proportional to the graph metric, global efficiency, at each node. We consider the patient P18 in state B in the reduced space (left part of the figure). We predict its changes when the *κ* index is decreased (point A) or increased (point C). Intuitively, point A and point C correspond respectively to a degradation and to an improvement of the health of the patient. Interestingly, these trends can be directly observed in the graph space for clinical insights (right part of the figure). It can be noticed that the variations are not linear.

Using the algorithm detailed in the last section, a map of the population is determined, with one of the reduced coordinates corresponding to a chosen covariate. To highlight the potential of the proposed method, we compute the transformation of a patient with regard to the changes of a covariate by creating a map from the reduced space back to the original space, where we can make brain-related interpretations. This gives us insight into the effect of the covariate on the patient. To perform this analysis, a regression is used to map the reduced space to the initial space (we used MARS regression [Friedman, 1991], coordinate-wise). This application is illustrated in Figure 8.

## DISCUSSION

### Assessment of Graph Metric Descriptors

The handcrafted design of graph metric descriptors is interesting since such descriptors carry straightforward physical meaning, like the *κ* index for hub reorganization. However, in a classification framework, such new scalar descriptors may not be optimal. To assess the pertinence of a new ad hoc graph metric descriptor for a classification task, it will be interesting to confront it with specific scalar coefficients used in standard classification algorithms (like LDA or FS), and examine whether there is some correlation between the scalars at stake. It is also worth mentioning that other approaches are dedicated to classifications directly using the connectivity matrices (Dadi et al., 2019; Richiardi et al., 2013). Usually, the objective of these approaches is to obtain classification scores, that is assign a subject to a group, patient, or control. To our knowledge, these approaches do not enable exploration of the underlying brain mechanisms at the nodal level using the results of classification (Ktena et al., 2018; Kumar et al., 2018; Yamin et al., 2019). Graph metrics are known to capture important topological indicators in networks that are impossible to capture using classical data-mining approaches (Zanin et al., 2016) or network embedding (Rosenthal et al., 2018).

### No Free Lunch for Graph Metric Descriptors

We hypothesize that there is no best descriptor adapted to all datasets. The optimality depends on (a) the kind of the data and (b) the kind of question/task addressed. This idea is known as the “no free lunch theorem” (Wolpert, 1996): If an algorithm performs well on a certain class of problems, then it has necessarily poorer performances on the set of all remaining problems.

In the present study, we showed that the *κ* index yields good classification performances in separating a comatose population from a healthy population. However, the MEAN index better describes the groups of elderly and young people (see Figure 3): For this dataset, the *κ* index cannot separate the two groups, but the MEAN score can. It is interesting to notice that several descriptors can correctly map a population, while providing different information.

The “no free lunch theorem” also applies to manifold learning algorithms. The underlying question is the one of choosing an interpoint distance in the data space. A given interpoint distance will yield a specific structure of samples in the reduced space. Therefore, the retained interpoint distance chosen will depend on the final goal, such as mimicking the structure of the initial data points, enhancing class separation with a view to achieving better classification performances, or focusing on a specific property of the data. The proposed algorithm CCML aims to mimic the structure of the initial data points by explicitly using a particular characteristic, chosen and imposed by the investigator.

### Manifold Learning for Brain Graph Analysis

Manifold learning is well suited for brain graph analysis for several reasons. First, global descriptors of graph metrics represent an entire graph by a scalar value, which is generally ultimately insufficient to correctly model the complexity of a graph population. Manifold learning is better suited to capture the complexity and variability of a given graph population, since more degrees of freedom are structuring the reduced space.

Second, connectomes have been studied for their capacity to represent the brain as a system and not merely as a juxtaposition of independent anatomical/functional regions. Classical statistical tests are not adapted to analyze joint variations between local descriptors of graph metrics since those tests assume independence between features. Brain graph manifold learning for comparing groups of graphs is promising because joint variations are accounted for.

Third, manifold learning may be turned to a generative model, when resorting to a mapping from the reduced space to the data space. Brain graph manifold learning can be seen as a trade-off between global and local brain graph metrics analysis. In other words, manifold learning is considered to be a model at the level of the group while preserving the information of the individuals. However, this technique is hard to interpret on its own. The addition of explicative covariables as proposed with the CCML method can provide an understandable and generative model of population with the possibility of focusing at the individual level (Costa et al., 2015). Using either global or local metrics is usually a hard task to appropriately link these features to clinical information. Statistical approaches suffer from a lack of interpretability where null and alternative hypotheses are tested. This is usually the case for coma studies where simple univariate statistical tests are computed on graph metrics (Demertzi et al., 2014; Malagurski et al., 2019). Using manifold learning, as illustrated in Figure 8, it is possible to provide a smooth description of the changes of the brain graphs of the patient in the reduced space. This approach can adequately relate the changes in brain connectivity along with the changes in clinical features. More generally, manifold learning can be an interesting solution for personalized and predictive medicine purposes. In our paper, we illustrate the result of our new proposed approach, CCML, on one graph metric, namely global efficiency. However, including several graph metrics is also a possibility and would lead to a more accurate description of the data, maybe at the cost of interpretability.

## CONCLUSION

The originality and contribution of this paper is the devising of a nonlinear manifold model of brain graph metrics. The essence of the approach is the capture of a metric through all nodes of a graph, across all graphs of an entire population. A population of graphs is represented by a population of vectors, each vector holding the variation of a metric through the nodes at stake. The population is then represented in a reduced space, as opposed to the standard representation of a given brain graph by a mere scalar.

The proposed approach has several advantages. First and foremost, the data are represented with several degrees of freedom, corresponding to the dimensions of the reduced space. The structure of the original dataset is captured by a compact representation. This allows us to account for the complex variability of populations of brain graphs. Second, such an approach naturally offers analysis of joint variations of those brain graph metrics. In addition, the investigator has the possibility to analyze the data at the population scale and simultaneously at the individual scale.

The investigation tool corresponding to the proposed approach allowed us to retrospectively assess the hub disruption index (HDI), denoted *κ*, and proposed in one of our former works. Earlier work showed that *κ* is a very good candidate for discriminating patients and controls in the case of coma. Retrospectively, its performance is assessed here in comparison with machine learning methods dedicated to linear group classification, such as LDA. Besides yielding nice classification performances, the present study showed that an advantage of *κ*, put in the perspective of a manifold model, is to give clinical clues related to the pathology mechanism.

We observed strong relationships between scalar coefficients such as *κ* and MEAN, and the coordinates of the manifold. It is important to notice that MEAN, which can separate groups in several pathologies (Lynall et al., 2010; Supekar et al., 2008), is not able to discriminate the comatose patients from the normal population. However, it brings additional information in terms of description of the population. The manifold at stake shows that a scalar coefficient cannot capture all of the information encapsulated in the graphs. One interesting aspect of manifold learning, and more specifically our new proposed method, is its ability to reach a new level of interpretation of the brain graph metrics and the interaction between them.

## ACKNOWLEDGMENTS

We want to thank the anonymous reviewers for their suggestions, which significantly improve the quality of our paper.

## SUPPORTING INFORMATION

Supporting information for this article is available at https://doi.org/10.1162/netn_a_00176. The algorithm is available at https://github.com/renardfe/CCML.

## AUTHOR CONTRIBUTIONS

Félix Renard: Conceptualization; Methodology; Software; Validation; Visualization; Writing Ű original draft; Writing Ű review & editing. Christian Heinrich: Conceptualization; Formal analysis; Methodology; Software; Supervision; Validation; Visualization; Writing Ű original draft; Writing Ű review & editing. Marine Bouthillon: Data curation; Formal analysis; Methodology. Maleka Schenck: Conceptualization; Data curation; Funding acquisition; Investigation; Resources. Francis Schneider: Conceptualization; Data curation; Funding acquisition; Investigation; Resources. Stéphane Kremer: Conceptualization; Data curation; Funding acquisition; Investigation; Project administration; Resources. Sophie Achard: Conceptualization; Formal analysis; Investigation; Methodology; Project administration; Software; Supervision; Validation; Visualization; Writing Ű original draft; Writing Ű review & editing.

## TECHNICAL TERMS

High-dimension, low-sample-size (HDLSS) framework: HDLSS is a mathematical concept to characterize a key challenge in data analysis, mainly the difference between the number of parameters and the number of observations.
ISOMAP methodology: ISOMAP is a dimension-reduction methodology consisting of projecting the initial observations to a reduced space, where it is simpler to describe and interpret the results.
*κ* or hub disruption index: *κ* is a graph metric to quantify the differences of locations of hubs and non-hubs between a patient and a group of controls.
