# Supplementary material for: A covariate-constraint method to map brain feature space into lower dimensional manifolds
Source: Netw Neurosci. 2021 Mar 1;5(1):252–73. doi: 10.1162/netn_a_00176 (PMC7935034; doi:10.1162/netn_a_00176)
Supplement: Supplementary file 1 [file netn-05-252-s001.pdf]

Renard, F., Heinrich, C., Bouthillon, M., Schenck, M., Schneider, F., Kremer, S. & Achard, S. (2021). Supporting information for "A covariateconstraint method to map brain feature space into lower dimensional manifolds." *Network Neuroscience*, 5(1), 252–273. https://doi.org/10.1162/netn_a_00176

A covariate-constraint method to map brain feature space into lower dimensional manifolds: Supporting Information

ELBOW CURVE

The choice for the dimension 2 of the reduced space takes into account mainly the ability to interpret the results. However, we checked also the curve of the error of the fitting, refelbow. This shows clearly that the dimension 2 is a good trade-off between minimisation of error and visualisation, it may be also worth exploring the dimension 3.

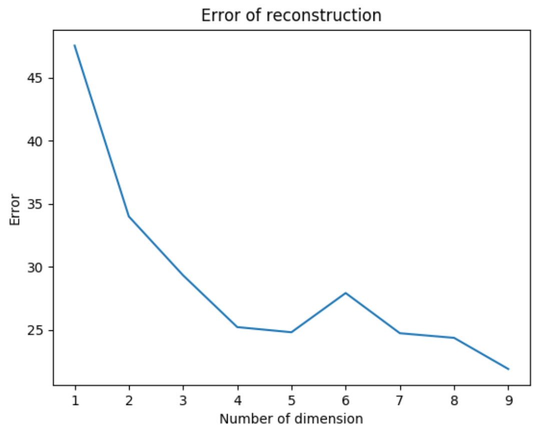

Figure 1. Elbow curve

Copyright ©2020 The Author(s). Published by MIT Press. All Rights Reserved.
